# Zinc-containing ionic liquid combined with carboxymethyl chitosan for vital pulp therapy in mature permanent teeth with carious pulp exposure

**DOI:** 10.1186/s12903-026-09392-2

**Published:** 2026-07-23

**Authors:** Zichao Zhou, Hongwei Chen, Chaoli Wang, Anya Liu, Minghao Wang, Chongyang Zhang, Li Fan, Yifang Yuan, Shengchao Wang, Wenkai Jiang

**Affiliations:** 1 State Key Laboratory of Oral & Maxillofacial Reconstruction and Regeneration, National Clinical Research Center for Oral Diseases, Shaanxi Key Laboratory of Stomatology, Department of Operative Dentistry and Endodontics, School of Stomatology, The Fourth Military Medical University, Xi’an, Shaanxi 710032 China; 2 Sanitation Unit of 75615 Unit of the Chinese People’s Liberation Army, Shenzhen, Guangdong 518110 China; 3 Department of Pharmacy, The Fourth Military Medical University, Xi’an, Shaanxi 710032 China; 4 The First Regiment, School of Basic Medicine, The Fourth Military Medical University, Xi’an, Shaanxi 710032 China; 5 Department of Stomatology, The 928th Hospital of PLA Joint Logistics Support Force, Haikou, Hainan 571159 China; 6 Medical Station of 73630 Unit of the Chinese People’s Liberation Army, Fuzhou, Fujian 350000 China

**Keywords:** Vital pulp therapy, Ionic liquid, Zinc, Carboxymethyl chitosan, Antimicrobial agent, Carious pulp exposure

## Abstract

**Background:**

The elimination of pulpal infection is critical for the success of vital pulp therapy (VPT) in mature permanent teeth with carious pulp exposure. However, commonly used pulp capping materials often possess inadequate antibacterial properties, which limits treatment success. This study aimed to develop a zinc-containing ionic liquid (IL-Zn)/carboxymethyl chitosan (CMCS) composite and evaluate its potential as an adjunctive pulp capping material for VPT.

**Methods:**

IL-Zn was characterized by energy dispersive spectroscopy (EDS), Fourier transform infrared spectroscopy (FTIR), proton nuclear magnetic resonance (^1^H NMR), and electrospray ionization mass spectrometry (ESI-MS). Its antibacterial activity against *Streptococcus mutans* (*S. mutans*) and *Lactobacillus casei* (*L. casei*) was assessed using minimum inhibitory concentration (MIC), minimum bactericidal concentration (MBC), time-killing curves, and scanning electron microscopy (SEM). The effect on dual-species biofilms was evaluated by crystal violet staining and live/dead staining. The potential for bacterial resistance to IL-Zn was examined through serial passage experiments. IL-Zn was then combined with CMCS to form the IL-Zn/CMCS composite. The antimicrobial efficacy of the composite against *S. mutans* and *L. casei* was tested by measuring the optical density of bacterial suspensions. The biocompatibility of the IL-Zn/CMCS composite was assessed via cell adhesion and SEM imaging. An animal model of pulpitis was used to evaluate the IL-Zn/CMCS composite as an adjunct to iRoot BP Plus for pulp capping.

**Results:**

EDS, FTIR, ^1^H NMR, ESI-MS collectively confirmed the successful synthesis of IL-Zn. In vitro antibacterial experiments demonstrated that IL-Zn, which exhibited a low propensity for inducing resistance, significantly inhibited both planktonic bacteria and dual-species biofilms. The IL-Zn/CMCS composite showed good antibacterial activity and biocompatibility with L929 cells. In animal tests, the composite improved pulp healing and dentin formation compared with iRoot BP Plus alone.

**Conclusions:**

This study developed an IL-Zn with strong antibacterial properties and a low risk of resistance. When combined with CMCS, the composite exhibited favorable antibacterial activity and biocompatibility. Used together with iRoot BP Plus for the treatment of carious pulp exposure in mature permanent teeth, it significantly enhanced pulp healing and dentin repair, offering a promising strategy to improve the success rate of vital pulp therapy.

**Supplementary Information:**

The online version contains supplementary material available at https://doi.org/10.1186/s12903-026-09392-2.

## Background

Dental pulp is essential for tooth health and longevity [1, 2]. In mature permanent teeth, carious exposure often leads to bacterial infection and pulp inflammation. Traditionally, root canal treatment (RCT) is recommended, but it involves pulp removal and reduction of tooth structure, potentially compromising tooth durability. Recently, vital pulp therapy (VPT) has gained popularity as a less invasive alternative, largely due to advances in pulp biology [3–5].

VPT aims to preserve pulp vitality by using biocompatible pulp‑capping materials to protect exposed or adjacent pulp tissue, prevent irritants, control infection, and promote dentin repair. The success of this treatment depends on the material’s ability to manage infection and support healing [2, 6]. Calcium silicate‑based pulp capping materials are widely used owing to their sealing properties, biocompatibility, mineralization‑promoting capacity, and inherent antibacterial activity [7, 8]. However, their antibacterial efficacy is often insufficient for treating pulpitis following carious exposure, leading to VPT failure [9, 10]. To address this limitation, studies have explored enhancing these materials with antimicrobial agents such as chlorhexidine (CHX). Nevertheless, these modification strategies have notable drawbacks, including the potential to induce bacterial resistance and compromise the sealing and biomineralization properties of the materials, which may adversely affect treatment outcomes [11–13]. Therefore, new antibacterial agents for VPT in mature permanent teeth with pulpitis following carious exposure are urgently needed. Such agents should minimize the risk of resistance while preserving the sealing and mineralization abilities of the pulp‑capping material.

In the search for innovative antibacterial agents, ionic liquids (ILs), particularly alkylimidazolium ILs, have attracted considerable attention due to their potent antibacterial activity, low propensity to induce drug resistance, excellent structural tunability, and favorable biocompatibility [14–16]. These characteristics make them promising candidates to overcome the limitations inherent in conventional antibacterial agents. The antibacterial mechanism of alkylimidazolium ILs involves the cationic component binding to the negatively charged bacterial surface, while the hydrophobic alkyl chains insert into the bacterial cell membrane, causing membrane disruption and subsequent cell death [17, 18]. This mode of action reduces the likelihood of resistance development—a common issue with traditional antibiotics—thereby providing a robust foundation for the application of alkylimidazolium ILs in VPT. However, to further enhance antibacterial efficacy, broaden the spectrum against potential pathogens in the complex pulp-capping environment, and reinforce the defense against resistance development, additional strategies are desirable. Zinc ions (Zn^2+^), recognized for their intrinsic antibacterial properties and ability to disrupt bacterial biofilms and metabolic pathways [19, 20], represent a compelling synergistic partner. We therefore synthesized a novel zinc-containing ionic liquid (IL-Zn) by integrating Zn^2+^ with alkylimidazolium ILs, aiming to achieve synergistic effects and superior antimicrobial activity relative to the IL alone. ILs inherently have high viscosity in their original state [21], which poses challenges for direct clinical operation. More importantly, when diluted to clinically applicable concentrations, IL-Zn becomes less viscous and tends to flow freely, making it difficult to localize and stably retain on the confined surface of exposed pulp tissue. To address these dual rheological limitations while preserving the anticipated antibacterial properties of IL-Zn, carboxymethyl chitosan (CMCS) was selected as a functional matrix. CMCS, a water-soluble chitosan derivative, offers excellent film-forming, adhesive, permeable, biocompatible, and biodegradable properties, rendering it highly suitable for formulating clinically feasible biomaterials with controlled release capabilities [22]. Crucially, the incorporation of CMCS is designed to preserve and enhance the antibacterial activity of IL-Zn.

This study aims to develop and characterize an innovative antimicrobial composite (designated IL-Zn/CMCS) by incorporating IL-Zn into a CMCS matrix. This design strategy offers a dual advantage: (1) the IL-Zn component provides potent, synergistic antibacterial activity with a reduced risk of inducing microbial resistance, and (2) the CMCS matrix facilitates practical clinical application by resolving the handling challenges associated with the viscosity of ILs, while preserving and potentially enhancing antibacterial efficacy. We propose that the application of the IL-Zn/CMCS composite as an adjunctive pulp-capping agent in VPT for mature permanent teeth with pulpitis resulting from carious exposure will significantly improve infection control and promote reparative dentin formation, thereby increasing treatment success rates.

## Methods

### Synthesis and characterization of IL-Zn

The synthesis of IL-Zn was based on previous studies [23]. A mixture of 1-vinylimidazole (0.05 mol, 4.7 g, Aladdin, Shanghai, China) and 1-bromododecane (0.05 mol, 12.2 g, Aladdin, Shanghai, China) was added to ethyl acetate (50 mL). The solution was purged with argon for 15 min to remove oxygen and magnetically stirred at 45 °C for 48 h. The resulting product was washed with ethyl acetate. The viscous liquid transformed into a white solid, which was dried under vacuum for 12 h to yield 1-vinyl-3-dodecylimidazolium bromide. This compound (0.03 mol, 9.9 g) was then reacted with anhydrous zinc chloride (0.03 mol, 4.1 g, Aladdin, Shanghai, China) in anhydrous methanol (30 mL) at room temperature for 24 h. After evaporating the methanol, the residue was dried to obtain 1-vinyl-3-dodecylimidazolium zinc chloride (denoted as IL-Zn). The IL-Zn was characterized via energy dispersive spectroscopy (EDS, Hitachi S-4800, Hitachi High-Technologies Corporation, Tokyo, Japan), Fourier transform infrared spectroscopy (FTIR, IRTracer-100, Shimadzu Corporation, Japan), Proton Nuclear Magnetic Resonance (^1^H NMR, Bruker Avance NEO 400, Bruker BioSpin GmbH, Rheinstetten, Germany), and electrospray ionization mass spectrometry (ESI-MS, Thermo Scientific Q Exactive, Thermo Fisher Scientific, USA). Subsequently, the IL-Zn was dissolved in double-distilled water (DDW) and maintained at 4 °C for future experimental applications.

### Bacterial culture

*Streptococcus mutans* (*S. mutans*) UA159 and *Lactobacillus casei* (*L. casei*) ATCC 393 were provided by the State Key Laboratory of Oral & Maxillofacial Reconstruction and Regeneration, School of Stomatology, the Fourth Military Medical University. These strains were selected because they are the predominant pathogens responsible for carious pulp exposure of mature permanent teeth [10, 24]. Before use, *S. mutans* was cultured overnight at 37 °C with 5% CO_2_ in Brain Heart Infusion (BHI, Haibo Biological Technology, Qingdao, China) broth, and *L. casei* was cultured under the same conditions in de Man, Rogosa and Sharpe (MRS, Haibo Biological Technology, Qingdao, China) broth.

### Minimum Inhibitory Concentration (MIC) and Minimum Bactericidal Concentration (MBC) determination

The antibacterial efficacy of the IL-Zn against *S. mutans* and *L. casei* was evaluated through MIC and MBC assay [25]. *S. mutans* and *L. casei* suspensions were diluted to 2 × 10^6^ CFU/mL, respectively, and then equal volumes (1:1 ratio) of the bacterial suspension and serially diluted IL-Zn solution (5.22 ~ 167.04 µg/mL) were added to 96-well plates. Sodium hypochlorite (NaClO, 102.5 ~ 3280 µg/mL) and CHX (2.44 ~ 78.08 µg/mL) served as positive controls, and phosphate-buffered saline (PBS) as the negative control (NC). Plates were incubated at 37 °C with 5% CO_2_ for 24 h. The MIC was the lowest concentration without visible bacterial growth (clear well without turbidity or precipitation). For MBC, 10 µL from clear wells were plated on BHI or MRS agar. After 48 h at 37 °C with 5% CO_2_, the MBC was the lowest concentration without colonies.

### Observation of bacterial morphology by Scanning Electron Microscopy (SEM)

*S. mutans* and *L. casei* suspensions were diluted to 2 × 10^6^ CFU/mL, respectively, and then thoroughly mixed at a 1:1 ratio. The mixed bacterial suspension was exposed to IL-Zn at a final concentration corresponding to its MIC. The mixture was incubated at 37 °C under 5% CO_2_ for 12 h, with PBS as the NC. After incubation, the bacteria were collected by centrifugation at 4000 rpm for 5 min, washed with PBS, and fixed overnight with 2.5% glutaraldehyde. Subsequently, the samples were dehydrated through a graded ethanol series in the following sequence: 30%, 50%, 70%, 80%, 90%, and 100% ethanol. Bacterial morphology was then observed using SEM (Hitachi S-4800, Hitachi High-Technologies Corporation, Tokyo, Japan).

### Time-dependent killing assays

The method used in this experiment was based on previous studies [26]. Bacterial suspensions were diluted to 1 × 10^6^ CFU/mL in broth containing test compounds or not, and incubated in 5% CO_2_ at 37 °C. The final concentration of IL-Zn, NaClO and CHX was equivalent to their MBC. At 0, 0.5, 1, 2, 4, 6, 8, 10, 12, 14, 16, 18, 20, 22, and 24 h, samples were serially diluted and 100 µL aliquots were plated on agar. The time-dependent killing curves were constructed by plotting the log_10_ (CFU/mL) versus the incubation time.

### Effect on dual-species biofilms evaluated by crystal violet staining

Crystal violet staining was performed to evaluate the effect of IL-Zn on *S. mutans* and *L. casei* dual-species biofilms. For dual-species biofilm formation inhibition: *S. mutans* and *L. casei* were diluted to 2 × 10^6^ CFU/mL in 1% BHIS (BHI supplemented with 1% (w/v) sucrose), and mixed equally. Then 100 µL of the mixed suspension was added to each well of 96-well plates with 100 µL of serially diluted IL-Zn (1.305 ~ 5.22 µg/mL). NaClO (410 ~ 1640 µg/mL) and CHX (2.44 ~ 9.76 µg/mL) were used as positive controls, and PBS as the NC. Plates were incubated at 37 °C with 5% CO_2_ for 24 h. After incubation, biofilms were fixed with 4% paraformaldehyde, stained with 0.1% (w/v) crystal violet (Biosharp, Hefei, China), and solubilized with 95% ethanol. The optical density (OD) of the solubilized dye was measured at 595 nm. For dual-species mature biofilm eradication: the same dual-species suspension was incubated for 24 h to form mature biofilms. Then, the supernatant was removed and 200 µL of serially diluted IL-Zn solution (2.61 ~ 41.76 µg/mL) was added to the preformed biofilms, with NaClO (205 ~ 820 µg/mL) and CHX (1.22 ~ 39.04 µg/mL) as positive controls, and plain BHI broth as the NC. After another 24 h incubation, biofilms were processed identically, and OD values were measured at 595 nm.

### Biofilm live/dead staining

*S. mutans* and *L. casei* were diluted to 2 × 10^6^ CFU/mL in 1% BHIS, mixed equally, and added to 24-well plates. The plates were then incubated at 37 °C with 5% CO_2_ for 24 h to form mature biofilms. Subsequently, BHI broth containing IL-Zn, NaClO, and CHX was added at a final concentration corresponding to the lowest concentration that eradicated the dual-species mature biofilm. BHI broth alone served as the NC. Plates were incubated for another 1 h. Post-incubation, the biofilms were stained using a Live/Dead assay kit (Beyotime Biotechnology, Shanghai, China) and observed using a confocal laser scanning microscope (CLSM, Olympus FV1000, Olympus Corporation, Tokyo, Japan). Quantitative analysis of the percentage of viable/dead bacteria within the biofilms was conducted by measuring mean fluorescence intensity.

### Bacterial drug resistance assay

To evaluate whether *S. mutans* and *L. casei* develope drug resistance against IL-Zn and CHX, serial passage experiments were performed [27]. The initial MIC of each agent against each bacterium was determined as described above. For each passage, 20 µL of bacterial suspension taken from the well with 1/2 MIC was mixed with 1,980 µL of fresh corresponding medium to eliminate residual drug. A volume of 50 µL of the resultant bacterial suspension was then spread onto BHI or MRS agar and incubated at 37 °C for 48 h. The colonies grown on the agar plate were resuspended in BHI or MRS broth. The overnight culture was then adjusted for the next MIC test. This procedure was repeated for 15 consecutive passages, and the MIC values were recorded at each passage to monitor any increase. A four-fold or greater increase in MIC compared to the initial MIC was considered indicative of acquired resistance.

### Preparation of IL-Zn/CMCS composites

IL-Zn was diluted to MIC with DDW and blended with CMCS to obtain composites with CMCS concentrations of 1%, 2%, 3%, 4%, 5%, 6% (w/v). Viscosity was measured using a rotational rheometer (MCR302e, Anton Paar GmbH, Graz, Austria).

### Antibacterial activity of IL-Zn/CMCS composites

IL-Zn/CMCS composites were prepared with serially diluted IL-Zn at 1.31 µg/mL, 2.61 µg/mL and 5.22 µg/mL, and CMCS at mass concentrations of 1%, 2%, 3%, 4%, 5% and 6% (w/v). Serial two-dimensional drug gradients were added into a 96-well plate at a volume of 100 µL per well following the checkerboard assay. Bacterial suspensions of *S. mutans* and *L. casei* were adjusted to 2 × 10^6^ CFU/mL, and 100 µL of each suspension was dispensed into corresponding wells. The plate was incubated at 37 °C for 24 h in a constant-temperature incubator. Well turbidity was assessed visually; the minimum drug concentrations showing no visible turbidity were defined as the MIC values for single or combined agents. FICI was calculated as: FICI = (MIC of IL-Zn in combination /MIC of IL-Zn alone) + (MIC of CMCS in combination / MIC of CMCS alone). The interaction between the two components was classified based on FICI values: synergism at FICI ≤ 0.5, additive effect at 0.5 < FICI ≤ 4, and antagonism at FICI > 4.

*S. mutans* and *L. casei* suspensions were diluted to 2 × 10^6^ CFU/mL, respectively. Then, 100 µL of the suspensions and 100 µL of IL-Zn/CMCS composites (CMCS at 1 ~ 6%, IL-Zn at 2.61 µg/mL) were added to each well of 96-well plates. The bacterial suspensions with PBS served as NC group. Plates were incubated at 37 °C with 5% CO_2_. After incubation for 24, 48, and 72 h, the OD value of each well was measured at 600 nm.

### Cell culture

The mouse fibroblast cell line (L929) was obtained from the American Type Culture Collection. L929 cells were cultured in complete Dulbecco’s modified Eagle medium (DMEM) supplemented with 10% fetal bovine serum and 1% penicillin-streptomycin at 37 °C with 5% CO_2_. The medium was renewed every two days.

### Cell adhesion

Cell adhesion assays were conducted according to previous research [28]. Uniformly coat 100 µL of 2% or 3% IL-Zn/CMCS onto the bottom of the wells in 12-well plates. Subsequently, seed L929 cells at a density of 2 × 10^5^ cells/well into the pre-coated wells. PBS served as the NC. After 24-hour incubation at 37 °C with 5% CO_2_, microscopic images were captured and cell counting was performed to calculate the cell adhesion rate. Cell morphology was examined using SEM.

### Animal experiment

The study protocol was approved by the Animal Ethics Committee of the School of Stomatology, Fourth Military Medical University (Approval No. 2024-kq-040). This study conformed to the ARRIVE guidelines.

Eighteen 10-week-old male Sprague-Dawley (SD) rats (300–380 g) with intact dentition were procured from the Experimental Animal Center of Fourth Military Medical University (License No. SCXK (Shaan) 2019-001). After a 2-day acclimatization period in individually ventilated cages (IVC) set at 22 ± 1 °C with 60–70% humidity and a 12-hour light/dark cycle (housing three rats per cage with unrestricted access to standard chow and sterile DDW), the animals were monitored throughout the study according to predefined humane endpoints, which included more than 10% body weight loss, severe lethargy, or inability to eat or drink.

Rats were randomly divided into three groups (*n* = 6): the BC group underwent pulp exposure followed by direct pulp capping with iRoot BP Plus (Innovative BioCeramix Inc., Vancouver, Canada) and restoration with light-cured glass ionomer cement (GIC, 3 M ESPE, St. Paul, MN, USA) without bacterial inoculation; the NC group experienced pulp exposure followed by the application of bacterial suspension, and then received iRoot BP Plus pulp capping and GIC restoration; the experimental group (IL-Zn/CMCS group) underwent the same bacterial inoculation as the NC group, but with the additional application of 2% IL-Zn/CMCS composite prior to pulp capping and restoration.

The rat pulpitis following carious exposure model was established according to previously described protocols [10]. The rats were anesthetized via intraperitoneal injection of 1% sodium pentobarbital at a dosage of 70 mg/kg. They were positioned in a supine position and subjected to oral disinfection using iodophor swabs. An FG 1/4 round bur was used to gain access to the pulp chambers of bilateral maxillary first molars via the occlusal central fossa, until a pinkish hue was visible, signifying close proximity to the pulp. Pulp perforation was completed using a #40 K-file. After irrigation with physiological saline and gentle drying with sterile cotton pellets, a mixed bacterial suspension containing 1 × 10^5^ CFU/mL each of *S. mutans* and *L. casei* was injected into the pulp chamber, followed immediately by direct pulp capping. The 4-week observation period was selected to comprehensively evaluate both the local inflammatory response and the formation of reparative dentin. All capping materials remained on the pulp wound surface throughout the 4-week observation period without dislodgement. All animals successfully completed the 4-week study period without attrition. To minimize confounding variables, daily cage rotation, randomized procedural sequencing, and blinded outcome assessments were employed.

At the end of the experiment, rats were euthanized via anesthetic overdose. Subsequently, the maxillae were dissected, fixed in 4% paraformaldehyde, decalcified in 10% EDTA, then processed into paraffin sections and stained with hematoxylin and eosin (H&E). Histological features, including inflammation cell infiltration, tissue disorganization, and reparative dentin formation, were evaluated and scored according to the histological scoring criteria (Table 1) [29]. Major organs, including the heart, liver, spleen, lungs, and kidneys, were similarly fixed in 4% paraformaldehyde and processed for H&E staining to assess potential systemic histopathological changes.

**Table 1:** Evaluation criteria for each observation item

Histological features	Grade	Evaluation criteria
Inflammation cell infiltration	0	No or only a few scattered inflammatory cells
	1	Mild inflammatory cell infiltration
	2	Moderate inflammatory lesions involving 1/3 of the radicular pulp
	3	Severe inflammatory lesions involving the entire radicular pulp
Pulp tissue disorganization	0	Essentially normal
	1	Disrupted and degenerated
	2	Pulp necrosis involving 1/3 of the radicular pulp
	3	Most of the pulp or complete necrosis of the radicular pulp
Reparative dentin formation	0	No dentin bridge formation
	1	A small amount of dentin bridge formation
	2	A moderate amount of dentin bridge formation
	3	A large amount of dentin bridge formation

### Statistical analysis

Data were analyzed using SPSS 26.0. Normally distributed data were assessed by one-way ANOVA with Dunnett’s post hoc test; non-parametric data used the Kruskal-wallis test followed by Dunn’s post hoc test (α = 0.05).

## Results

### Characterization of IL-Zn

**Fig. 1:**
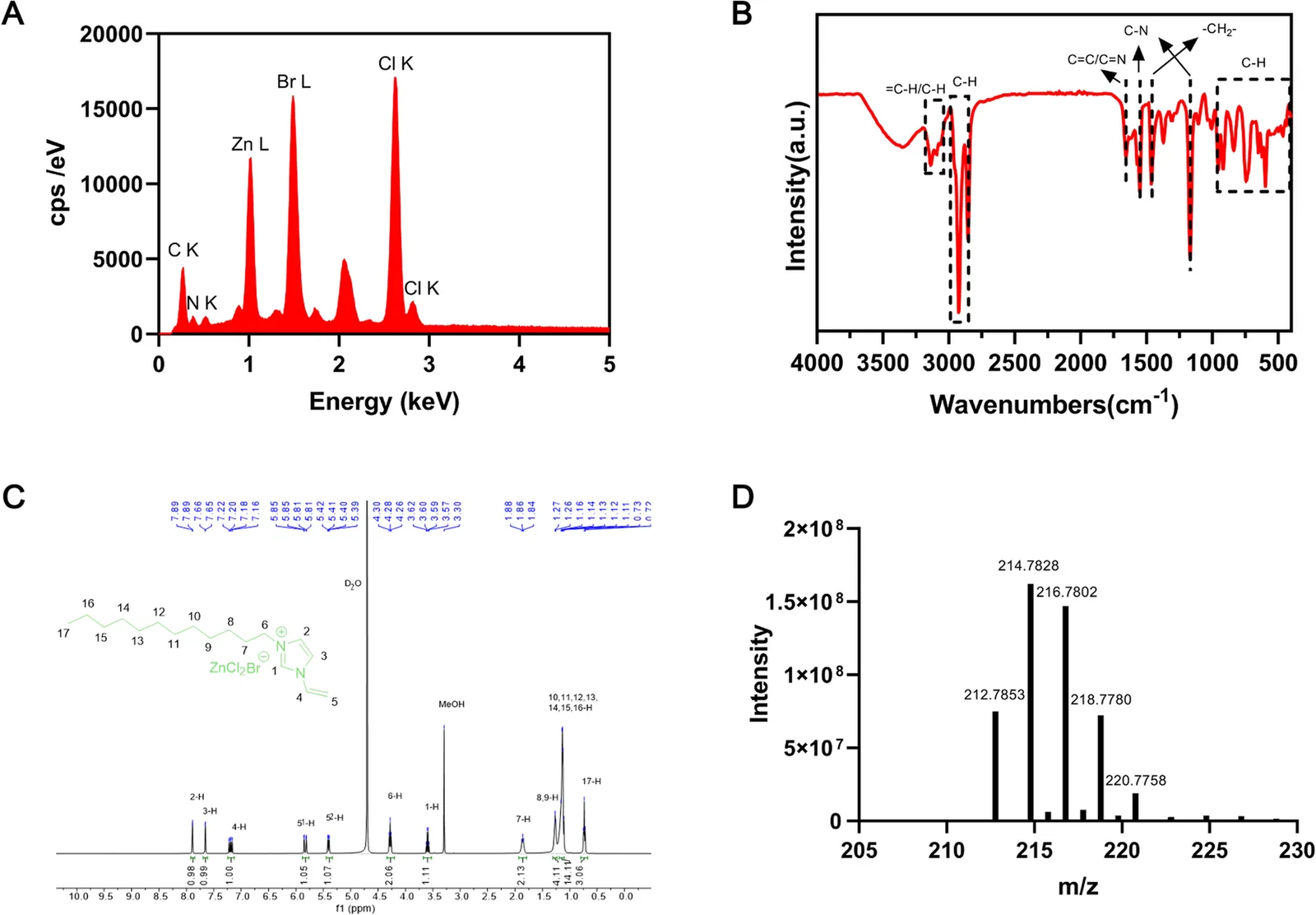
Material characterization results of IL-Zn. **A** EDS spectra of IL-Zn. **B** FTIR spectra of IL-Zn. **C** ^1^H NMR Spectrum of IL-Zn. **D** ESI-MS of IL-Zn

EDS analysis (Fig. 1A) revealed that the main elemental peaks were C, N, Br, Cl, and Zn, which was consistent with the elemental composition of IL-Zn. The FTIR spectra (Fig. 1B) showed peaks at: 3100–3200 cm^− 1^ (vinyl C-H stretching and aromatic C-H stretching in the imidazole ring), 2850–2950 cm^− 1^ (C-H stretching in the dodecyl group and imidazole), 1654 cm^− 1^ (C = C and C = N vibrations in imidazole), 1549 cm^− 1^ and 1168 cm^− 1^ (C-N stretching), and 1461 cm^− 1^ (-CH_2_- scissoring in the alkyl chain). These spectral characteristics were consistent with the molecular structure of IL-Zn.

As shown in Fig. 1C, the structure of the synthesized compound was confirmed by ^1^H NMR spectroscopy. The characteristic signals were as follows: the imidazolium ring protons appeared at δ 7.89 (d, J = 2.1 Hz, 1H, H-2), and δ 7.66 (d, J = 2.1 Hz, 1H, H-3). The vinyl group (-CH=CH_2_) exhibited signals at δ 7.19 (dd, J = 15.6, 8.7 Hz, 1H, H-4), δ 5.83 (dd, J = 15.6, 2.9 Hz, 1H, H-5a), and δ 5.41 (dd, J = 8.7, 2.9 Hz, 1H, H-5b). The methylene group directly attached to the imidazolium ring (-N-CH_2_-) was observed at δ 4.28 (t, J = 7.2 Hz, 2 H, H-6). The long alkyl chain gave signals at δ 1.86 (t, J = 7.2 Hz, 2 H, H-7), δ 1.26 (d, J = 5.1 Hz, 4 H, H-8,9), δ 1.21–1.07 (m, 14 H, H-10-16), and δ 0.80 − 0.65 (m, 3 H, H-17). These assignments are consistent with the expected structure of IL-Zn. ESI-MS results (Fig. 1D) showed equidistant peaks at m/z 212.7853, 214.7828, 216.7802, 218.7780, and 220.7758. Combined with the stable isotopic patterns of chlorine (^35^Cl/^37^Cl), bromine (^79^Br/^81^Br), and zinc (^64^Zn/^66^Zn), these signals were identified as correspond to the [ZnCl_2_Br]^−^. These evidence corroborated the successful synthesis of IL-Zn.

### Antibacterial activity of IL-Zn

**Fig. 2:**
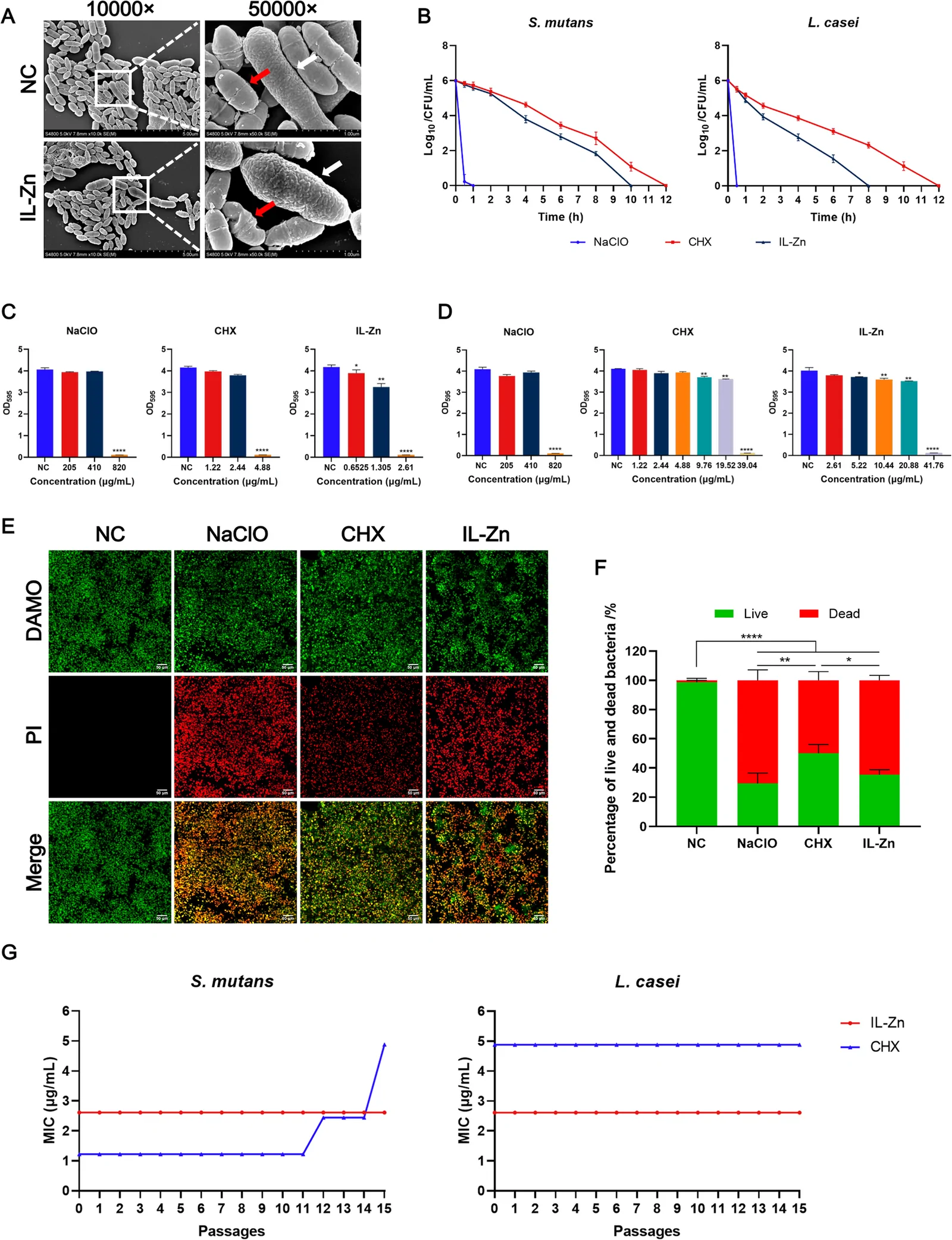
Antibacterial activity of IL-Zn. **A** Representative SEM images of morphology of *S. mutans* (red arrows) and *L. casei* (white arrows). Bar scale, 5 μm and 1 μm. **B** Time-killing curves of different materials against planktonic *S. mutans* and *L. casei*. **C** Effects of different treatments on dual-species biofilm formation. **D** Effects of different treatments on dual-species mature biofilm eradication. **E** Representative CLSM images of dual-species biofilm after different treatments. Bar scale, 50 μm. **F** Percentage of live and dead bacteria in dual-species biofilm after different treatments. **G** MICs of IL-Zn and CHX against planktonic *S. mutans* and *L. casei* after repeated exposure from passages 0 to 15. NC: negative control group. Values are presented as mean ± SD (*n* = 3). All experiments were performed three times. *: *p* < 0.05, **: *p* < 0.01, ****: *p* < 0.0001

To evaluate the antibacterial activity of IL-Zn, the MIC and MBC assays were carried out. IL-Zn demonstrated antimicrobial efficacy comparable to CHX. Against *S. mutans*, the MIC and MBC of IL-Zn were 2.61 µg/mL and 10.44 µg/mL, respectively; against *L. casei*, the MIC and MBC were 2.61 µg/mL and 41.76 µg/mL, respectively (Table 2). SEM showed that IL-Zn treatment at the MIC caused significant morphological changes in *S. mutans* and *L. casei*, resulting in shrinkage and deformation, while the NC group maintained regular morphology (Fig. 2A). As illustrated in Fig. 2B, the time-kill curves showed that at their respective MBCs, the viable count of *S. mutans* decreased rapidly after 0.5 h of NaClO treatment and was completely inactivated at 1 h; complete killing was achieved at 12 h for CHX and at 10 h for IL-Zn. For *L. casei*, NaClO induced total bacterial death after 0.5 h of treatment, whereas CHX and IL-Zn achieved complete killing at 12 h and 8 h, respectively. Crystal violet staining results (Fig. 2C, D) demonstrated that 820 µg/mL NaClO, 4.88 µg/mL CHX, and 2.61 µg/mL IL-Zn completely inhibited the formation of dual-species biofilms of *S. mutans* and *L. casei*, with statistically significant differences compared with the NC group (*p* < 0.0001). Furthermore, 820 µg/mL NaClO, 39.04 µg/mL CHX, and 41.76 µg/mL IL-Zn completely eradicated the preformed dual-species biofilms of *S. mutans* and *L. casei*, and the differences were also statistically significant relative to the NC group (*p* < 0.0001). Live/dead staining (Fig. 2E, F) assessed the effectiveness of three antimicrobial agents (820 µg/mL NaClO, 39.04 µg/mL CHX, and 41.76 µg/mL IL-Zn) against mature dual-species biofilms after 1 h. The live cell proportions were 29.4% for NaClO, 50.1% for CHX, and 35.6% for IL-Zn. Statistical analysis indicated that IL-Zn’s effectiveness was similar to NaClO (*p* > 0.05) but significantly higher than CHX (*p* < 0.05).

To investigate whether the two bacterial species developed resistance against IL-Zn, serial passage experiments were performed, and the results were shown in Fig. 2G. After 15 consecutive passages, the MIC of IL-Zn against both bacteria remained 2.61 µg/mL, indicating no development of bacterial resistance. In contrast, the MIC of CHX against *S. mutans* increased fourfold after 15 consecutive passages, indicating the emergence of resistance.

**Table 2:** MIC and MBC of IL-Zn against *S. mutans* and *L. casei*

Bacterial species	IL-Zn (µg/mL)		CHX (µg/mL)		NaClO (µg/mL)	
	MIC	MBC	MIC	MBC	MIC	MBC
*S. mutans*	2.61	10.44	1.22	4.88	820	820
*L. casei*	2.61	41.76	4.88	39.04	820	820

### Antibacterial activity and cytocompatibility of IL-Zn/CMCS composites

**Fig. 3:**
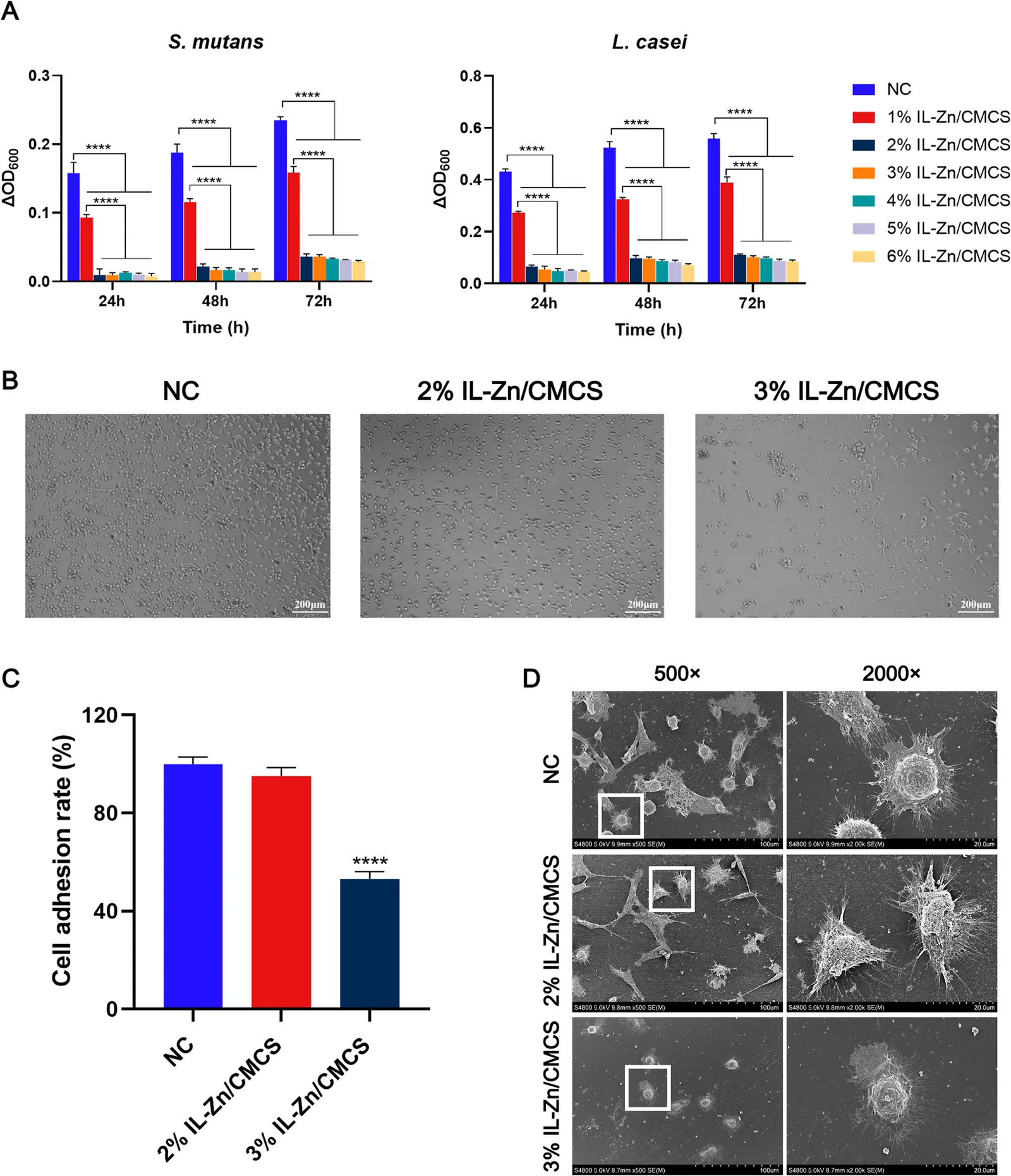
Antibacterial activity and cytocompatibility of IL-Zn/CMCS composites. **A** ΔOD_600_ of *S. mutans* and *L. casei* after treatment with varying concentrations of IL-Zn/CMCS composites for 24, 48, and 72 h. **B** Representative microscopy images of L929 cells adhering to substrates. Bar scale, 200 μm. **C** Quantitative analysis of cell adhesion rates. **D** Representative SEM images of morphology of adherent cell. Bar scale, 100 μm and 20 μm. NC: negative control group. Values are presented as mean ± SD (*n* = 3). All experiments were performed three times. ****: *p* < 0.0001

The physical appearance of 1 ~ 6% (w/v) IL-Zn/CMCS composites at room temperature was depicted in Fig. S1. A progressive deepening of color can be observed corresponding to increasing concentration. The viscosity of IL-Zn/CMCS composites exhibited a positive correlation with the concentration of CMCS; specifically, at a concentration of 4%, the viscosity escalated dramatically to 1027.3 ± 3.2 mPa·s, resulting in a marked reduction in flow rate, which adversely affected both spreadability and operability (Table S1). The combinational antibacterial interaction between IL-Zn and CMCS was evaluated via fractional inhibitory concentration index (FICI) assay. The MIC values of single-agent CMCS and IL-Zn against *S. mutans* were 3% and 2.61 µg/mL, respectively. When used in combination, the mixture containing 1% CMCS and 1.31 µg/mL IL-Zn achieved obvious antibacterial activity (Table S2). The same results were observed for *L. casei* (Table S3). The FICI values for both *S. mutans* and *L. casei* were 0.83, confirming an additive antibacterial effect of the two components (Table S4). Subsequently, a 72 h incubation test was performed to assess long-term antibacterial stability of the composites. As shown in Fig. 3A, all concentrations of IL-Zn/CMCS (CMCS at 1 ~ 6%, IL-Zn at 2.61 µg/mL) composite significantly inhibited the growth and proliferation of *S. mutans* and *L. casei* within 72 h, with statistically significant differences compared with the NC group (*p* < 0.0001). Furthermore, no statistically significant difference was observed in the antibacterial efficacy among the IL-Zn/CMCS composites at concentrations of 2% and above (*p* > 0.05). Cell adhesion studies demonstrated a substantial presence of healthy L929 cells on both the NC and 2% IL-Zn/CMCS composite surfaces (Fig. 3B). Conversely, the 3% IL-Zn/CMCS composite group exhibited a reduced cell count, with some cells appearing shrunken. Quantitative analysis (Fig. 3C) showed over 95% adhesion on the 2% IL-Zn/CMCS composite surface, with no significant difference from the NC (*p* > 0.05). In contrast, adhesion on the 3% IL-Zn/CMCS composite surface dropped to about 50%, showing a significant difference (*p* < 0.0001). SEM analysis (Fig. 3D) revealed that L929 cells on the 2% IL-Zn/CMCS composite surface exhibited a typical elongated spindle-shaped morphology as control group, with a high degree of cell spreading and abundant filopodia, indicating good cell adhesion. In contrast, the overall spreading of L929 cells on the 3% IL-Zn/CMCS composite surface was markedly inferior to that of the 2% IL-Zn/CMCS group, and the cell number was significantly reduced. This confirmed that 2% IL-Zn/CMCS possessed excellent cytocompatibility.

### Evaluation of dental pulp healing and reparative dentin formation in rats

**Fig. 4:**
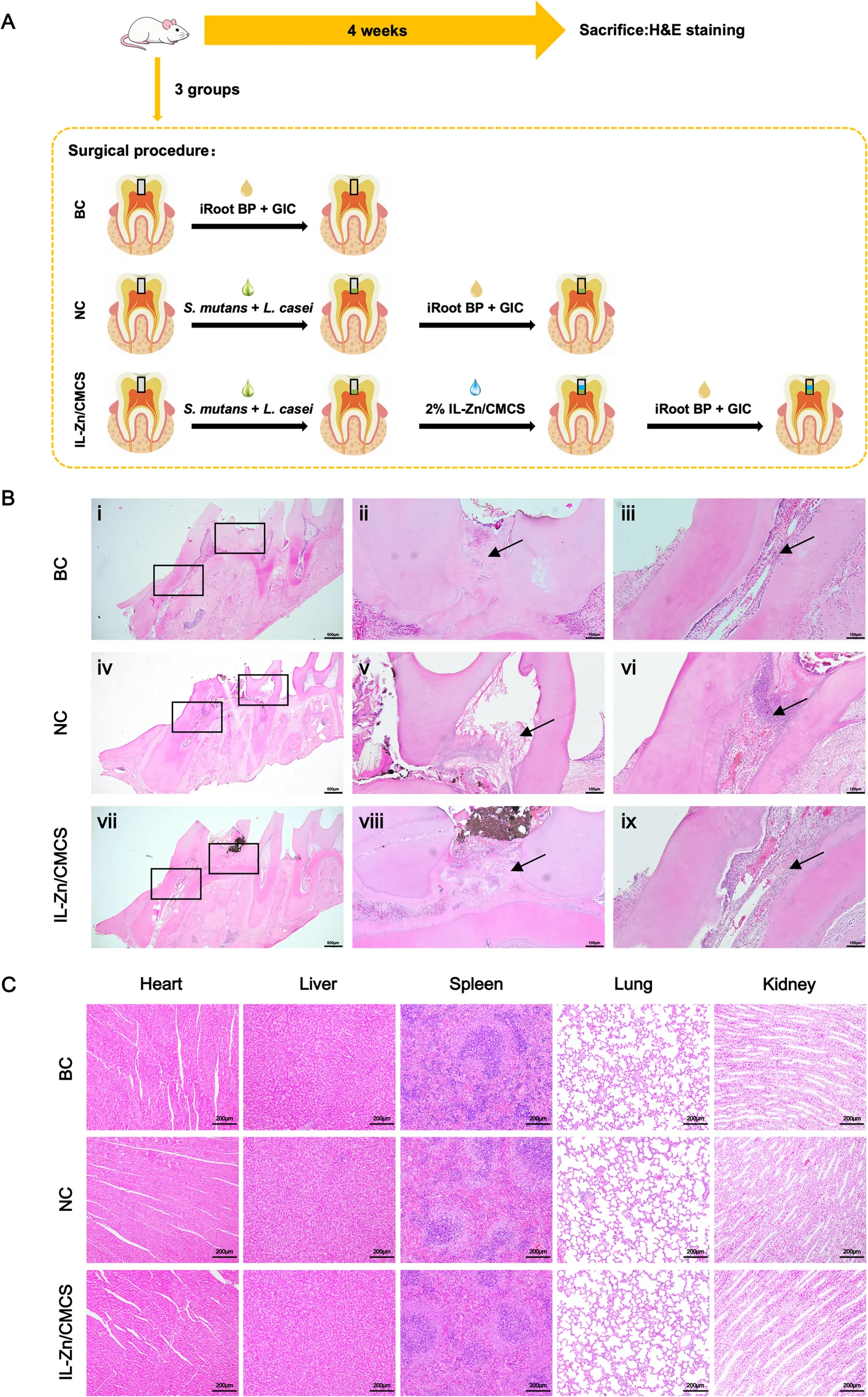
Dental pulp healing and reparative dentin formation in different groups in vivo. **A** Schematic diagram of animal experimental design. **B** Representative H&E staining of dental pulp after different treatments: (ii) Reparative dentin formation in the coronal pulp chamber (black arrow), (iii) Mild inflammatory infiltration in the radicular pulp (black arrow); (v) Focal pulp necrosis in the coronal pulp chamber (black arrow), (vi) Severe inflammatory infiltration in the radicular pulp (black arrow); (viii) Reparative dentin formation in the coronal pulp chamber (black arrow), (ix) Moderate inflammatory infiltration in the radicular pulp (black arrow). Bar scale, 500 μm and 100 μm. **C** Representative H&E staining images of tissues of various organs. Bar scale, 200 μm

The schematic diagram of the animal experimental design was shown in Fig. 4A. The H&E staining results (Fig. 4B) demonstrate that distinct reparative responses occurred across different groups. In the BC group, reparative dentin bridges formed beneath the pulp capping material with confined tissue destruction and mild inflammation. The NC group demonstrated partial to complete necrosis in the coronal pulp chamber, accompanied by severe inflammatory infiltration extending to the apical third of the radicular pulp. Minimal or disorganized reparative dentin deposition was observed adjacent to the capping material. In the 2% IL-Zn/CMCS composite combined with iRoot BP Plus group (hereafter referred to as the “IL-Zn/CMCS group”), reparative dentin deposition was observed beneath the pulp capping material, despite focal necrosis and mild inflammation at the pulp wound interface. Histological scores are detailed in Table 3. For inflammatory cell reaction, the BC group exhibited significantly lower inflammation scores (*p <* 0.05), characterized by scattered inflammatory cells or focal mild infiltration. This differed statistically from both the NC and IL-Zn/CMCS groups, whereas no significant difference existed between the NC and IL-Zn/CMCS groups (*p >* 0.05). Regarding tissue destruction, the IL-Zn/CMCS group demonstrated predominant degenerative changes and architectural disruption subjacent to the pulp wound interface. This was statistically distinct from the NC group (*p <* 0.05) but showed no significant difference compared to the BC group (*p >* 0.05). In reparative dentin formation, the NC group displayed minimal or disorganized reparative dentin deposition beneath the capping material, differing significantly from both the BC and IL-Zn/CMCS groups (*p <* 0.05). Conversely, the BC group and IL-Zn/CMCS group showed comparable reparative dentin bridge formation with no statistical difference (*p >* 0.05). No abnormalities were observed in major organs via H&E staining (Fig. 4C).

**Table 3:** Pulpal histological scores in each group

Group	*n*	Inflammatory infiltration					Tissue disruption					Reparative dentin formation				
		0	1	2	3	Md	0	1	2	3	Md	0	1	2	3	Md
BC	12	4	6	1	1	1^a^	4	6	1	1	1^a^	1	2	6	3	2^a^
NC	12	0	1	6	5	2^b^	0	3	7	2	2^b^	7	2	3	0	0^b^
2% IL-Zn/CMCS	12	0	4	5	3	2^b^	2	7	3	0	1^a^	0	2	7	3	2^a^

The groups with the same label letters have no significant difference

## Discussion

The complete elimination of pulpal infection is essential for the successful implementation of VPT in mature permanent teeth with carious pulp exposure [30]. However, existing pulp capping materials demonstrate inadequate antibacterial properties [31, 32], and traditional antimicrobial modification approaches pose the risk of promoting bacterial resistance, thereby significantly constraining the success rates of VPT. To address this issue, we have engineered an innovative IL-Zn by integrating alkylimidazolium ILs, recognized for their potent antibacterial properties, minimal likelihood of inducing drug resistance, exceptional structural tunability, and favorable biocompatibility, with Zn^2+^, which provide broad-spectrum antimicrobial effects. Subsequently, IL-Zn was incorporated into CMCS to form an antimicrobial composite (IL-Zn/CMCS). We evaluated the potential of this novel composite as an adjunctive agent for enhancing VPT outcomes.

In vitro investigations have demonstrated that IL-Zn exhibits significant inhibitory effects against both planktonic cells and dual-species biofilms of *S. mutans* and *L. casei*, which are principal pathogens in carious pulp exposure of mature permanent teeth [10, 24]. IL-Zn exhibited antimicrobial activity against *S. mutans* and *L. casei* comparable to that of CHX; at significantly reduced concentrations, it achieved complete dual-species biofilm inhibition and preformed biofilm eradication equivalent to CHX, whereas NaClO required substantially higher concentrations for comparable effects. This potent antibacterial activity might be attributed to the synergistic interaction between imidazolium ILs and Zn^2+^. According to the previous report [33], imidazolium cations can insert into and disrupt bacterial cell membranes, thereby forming permeability channels that lead to cytoplasmic leakage. Concurrently, such membrane damage facilitates the intracellular entry of Zn^2+^, which has been demonstrated to induce oxidative damage to bacterial DNA, enzyme systems, and proteins. Therefore, we hypothesize that the two components may act synergistically via a combined mechanism involving “membrane disruption and intracellular oxidative stress” to inhibit bacterial growth and proliferation. Notably, experiments designed to induce bacterial drug resistance further substantiated the advantages of IL-Zn. When *S. mutans* was continuously exposed to sub-inhibitory concentrations of the antimicrobial agent and subcultured for 15 generations, the MIC of CHX against this bacterium increased fourfold, indicating that CHX can induce bacterial drug resistance. This result is consistent with previous reports [34]. Conversely, the MIC for the IL-Zn group remained constant, demonstrating that IL-Zn is less prone to inducing bacterial drug resistance. This finding provides crucial experimental evidence for the long-term and safe management of pulp infections in clinical practice.

To ensure precise delivery and prolonged retention of IL-Zn on the pulp wound surface, this study integrated IL-Zn with CMCS, a material renowned for its excellent biocompatibility, to develop the IL-Zn/CMCS composite. CMCS is distinguished by its superior film-forming capabilities, tissue adhesiveness, and biodegradability, which facilitate the formation of a uniformly covering film on the pulp wound surface by the composite [35]. This film functions as a physical barrier, protecting the pulp tissue from exogenous stimuli during the initial stages of pulp capping, while also maintaining the local concentration of the antibacterial agent through a controlled-release mechanism [36].

Pure IL-Zn exhibits high viscosity and poor clinical spreadability [21]. Even after dilution to working concentrations, it shows insufficient retention on pulp tissues. To address these limitations, CMCS was incorporated to improve the overall handling performance. A FICI assay verified an additive antibacterial interaction between IL-Zn and CMCS, with no antagonistic effect, preliminarily indicating that CMCS does not compromise the antibacterial activity of IL-Zn.

Notably, the composite exhibited significant antibacterial activity against *S. mutans* and *L. casei* within 72 h at a concentration of 2% (w/v) and above. Such favorable antibacterial performance further supports that CMCS serves as a suitable carrier. It optimizes the handling properties of IL-Zn and confers a modest enhancement in antibacterial activity, consistent with our initial design intent.

To assess the in vivo efficacy of the composite, a rat pulpitis model following carious exposure was established using the maxillary first molars of 10-week-old SD rats. Previous studies have demonstrated that hydraulic calcium silicate cements not only exhibit favorable cytocompatibility, but also induce the formation of hydroxyapatite-like mineralized layers and promote the odontogenic differentiation of human dental pulp stem cells [37]. Accordingly, iRoot BP Plus was selected as the pulp-capping material in this study. The findings indicated that under conditions of bacterial infection, the application of iRoot BP Plus alone (NC group) resulted in minimal reparative dentin formation and was associated with significantly greater pulp inflammation and tissue destruction compared to the BC group (*p* < 0.05). This observation is consistent with previous reports [10]. Conversely, pretreatment with the 2% IL-Zn/CMCS composite prior to capping markedly reduced pulp tissue damage and significantly enhanced reparative dentin formation compared with NC group, achieving results comparable to those of the BC group. This underscores the strategic advantage of utilizing IL-Zn/CMCS-assisted pulp capping. Importantly, the study demonstrated that the integrity of the hermetic seal between iRoot BP Plus and dental tissues was maintained, and the material’s intrinsic ability to promote mineralization was not compromised in the IL-Zn/CMCS group, thereby affirming the clinical viability of this approach. Simultaneously, in vitro safety assessments and in vivo histopathological analyses of major organs validated excellent biocompatibility of the composite, providing a robust safety foundation for clinical application.

The present study provides preliminary evidence that IL‑Zn/CMCS exhibits potent antibacterial activity and favorable biocompatibility, and can serve as an effective adjunct to iRoot BP Plus to improve the success rate of vital pulp therapy. This novel material offers a new clinical strategy for vital pulp treatment. Future work will focus on comprehensive characterization and further optimization of IL‑Zn/CMCS to strengthen the preclinical evidence base for clinical translation. Long‑term monitoring will be conducted to assess its sustained antibacterial activity and its low propensity to induce bacterial resistance. Additional cellular studies are required to investigate the effects of IL‑Zn/CMCS on the proliferation, the odontogenic differentiation, and the mineralization of human dental pulp cells, and to clarify the underlying mechanisms.

## Conclusions

This study developed an IL-Zn with strong antibacterial properties and a low propensity for inducing bacterial resistance. Incorporation of IL-Zn into the CMCS matrix confers the composite with optimized clinical handling properties and moderately enhanced antibacterial activity, as verified by FICI results, while maintaining favorable biocompatibility. Animal tests revealed that the use of this composite together with iRoot BP Plus effectively promoted pulp tissue healing and reparative dentin formation in vital pulp therapy for mature permanent teeth with carious pulp exposure. These results suggest a promising new approach for improving the success rate of vital pulp preservation in mature permanent teeth affected by carious pulp exposure.

## Supplementary Information

Supplementary Material 1.

## Data Availability

The datasets used and/or analyzed during the current study available from the corresponding author on reasonable request.

## Abbreviations

RCT: Root canal treatment
VPT: Vital pulp therapy
CHX: Chlorhexidine
ILs: Ionic liquids
CMCS: Carboxymethyl chitosan
IL-Zn: Zinc-containing ionic liquid; 1-vinyl-3-dodecylimidazolium zinc chloride
FTIR: Fourier transform infrared spectroscopy
EDS: Energy dispersive spectroscopy
ESI-MS: Electrospray ionization mass spectrometry
DDW: Double-distilled water
MIC: Minimum inhibitory concentration
MBC: Minimum bactericidal concentration
*S. mutans*: *Streptococcus mutans*
*L. casei*: *Lactobacillus casei*
BHI: Brain Heart Infusion
MRS: De Man, Rogosa and Sharpe
NaClO: Sodium hypochlorite
PBS: Phosphate-buffered saline
ROS: Reactive oxygen species
SEM: Scanning electron microscopy
1% BHIS: BHI broth supplemented with 1% (w/v) sucrose
OD: Optical density
CLSM: Confocal laser scanning microscope
DMEM: Dulbecco’s modified Eagle medium
CCK-8: Cell counting kit-8
SD: Sprague-Dawley
IVC: Individually ventilated cages
GIC: Glass ionomer cement
EDTA: Ethylenediaminetetraacetic acid
H&E: Hematoxylin and eosin
