# Supplementary material for: Zinc-containing ionic liquid combined with carboxymethyl chitosan for vital pulp therapy in mature permanent teeth with carious pulp exposure
Source: BMC Oral Health. 2026 Jul 23;26:2056. doi: 10.1186/s12903-026-09392-2 (PMC13678887; doi:10.1186/s12903-026-09392-2)
Supplement: Supplementary file 1 [file 12903_2026_9392_MOESM1_ESM.docx]

**Supplementary Material**

**Fig. S1** Room-temperature physical appearance of IL-Zn/CMCS composites at graded concentrations. Samples i-vi correspond to 1%~6% (w/v) IL-Zn/CMCS composites, respectively.

**Table S1** Rheological properties of IL-Zn/CMCS composites at varying concentrations.

| IL-Zn/CMCS（w/v） | Viscosity (mPa·s) | Flowability |
| --- | --- | --- |
| 1% | 12.05±0.15 | Fast flow |
| 2% | 70.48±0.12 | Moderate flow |
| 3% | 299.2±1.6 | Slow flow |
| 4% | 1027.3±3.2 | Very slow flow |
| 5% | 2830±8 | Very slow flow |
| 6% | 6158±12 | Very slow flow |

**Table S2** Combined antibacterial efficacy of IL-Zn and CMCS against *S. mutans*

| agents and concentrations | | IL-Zn (µg·mL^-1^) | | | CMCS  alone |
| --- | --- | --- | --- | --- | --- |
|  |  | 1.31 | 2.61 | 5.22 |  |
| CMCS  (w/v) | 1% | + | + | − | + |
|  | 2% | + | − | − | + |
|  | 3% | + | − | − | + |
|  | 4% | + | − | − | + |
|  | 5% | + | − | − | + |
|  | 6% | − | − | − | − |
| IL-Zn alone | | + | + | − |  |

Note: “+” indicates visible turbidity, while “−” denotes no turbidity.

**Table S3** Combined antibacterial efficacy of IL-Zn and CMCS against *L. casei*

| agents and concentrations | | IL-Zn (µg·mL^-1^) | | | CMCS  alone |
| --- | --- | --- | --- | --- | --- |
|  |  | 1.31 | 2.61 | 5.22 |  |
| CMCS  (w/v) | 1% | + | + | − | + |
|  | 2% | + | − | − | + |
|  | 3% | + | − | − | + |
|  | 4% | + | − | − | + |
|  | 5% | + | − | − | + |
|  | 6% | − | − | − | − |
| IL-Zn alone | | + | + | − |  |

Note: “+” indicates visible turbidity, while “−” denotes no turbidity.

**Table S4** FICI values of IL-Zn combined with CMCS against *S. mutans* and *L. casei*

| Bacteria | MIC in combination/MIC alone | | FICI | Interaction |
| --- | --- | --- | --- | --- |
|  | CMCS (w/v) | IL-Zn (µg·mL^-1^) |  |  |
| *S. mutans* | 1/3 | 1.31/2.61 | 0.83 | additive effect |
| *L. casei* | 1/3 | 1.31/2.61 | 0.83 | additive effect |
